# Hybrid Semimagnetic Polaritons in a Strongly Coupled Optical Microcavity

**DOI:** 10.1021/acs.jpclett.1c01894

**Published:** 2021-08-05

**Authors:** Tomasz Fąs, Maciej Ściesiek, Wojciech Pacuski, Andrzej Golnik, Jan Suffczyński

**Affiliations:** Institute of Experimental Physics, Faculty of Physics, University of Warsaw, 5 Pasteura Street, 02-093 Warsaw, Poland

## Abstract

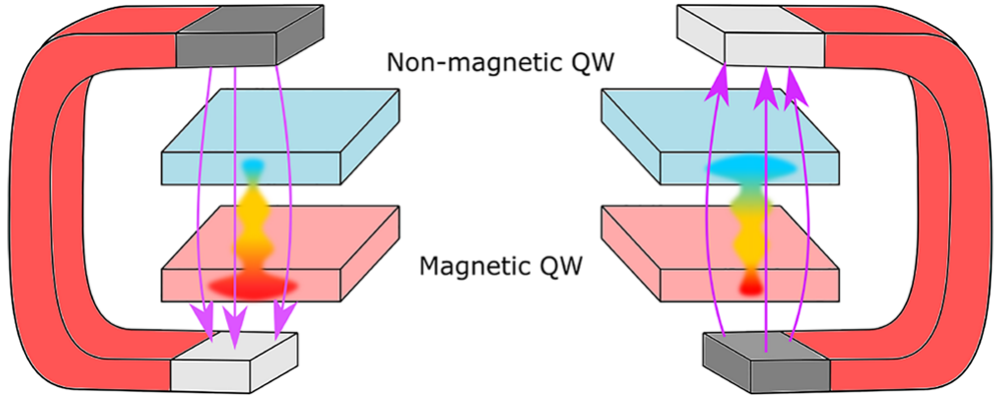

Exciton–polaritons of a hybrid type, emerging in a structure comprising semimagnetic (Mn-doped) and nonmagnetic quantum wells coupled via the microcavity optical mode are demonstrated and studied. Thanks to the susceptibility of the excitons in the magnetic quantum well to the magnetic field, all the emerging hybrid polariton states acquire magnetic properties. In that way, external magnetic field enables control over the degree of hybridization, tuning of the ratio of the excitonic to photonic components of the hybrid polaritons, and alteration of the direction and dynamics of the energy transfer between the excitonic states in magnetic and nonmagnetic quantum wells. The presented possibility of the hybridization of a semimagnetic exciton with an exciton in a material that itself does not exhibit any meaningful magnetic effects is highly promising in the context of the fabrication of—to date lacking—organic, perovskite, or dichalcogenide-based systems with strong magnetooptical properties.

A polariton is a quasiparticle combining properties of light and matter, most typically composed of an excitonic state strongly coupled to an optical mode of a microcavity.^1^ When more than one excitonic state couples to the same optical mode, a *hybrid* polariton state emerges (see also the Supporting Information). So far, such multicomponent polaritons have been realized in single^2−11^ or coupled^12−16^ microcavities incorporating spatially separated semiconductor layers. Hybridization has been shown to enable electrical control over polariton composition,^7^ nonlinearities engineering,^17^ or polariton-mediated energy transfer over micrometer-range distances.^11,14,15^

In recent years, a new class of polaritons has arisen, called *semimagnetic* polaritons,^18,19^ which are formed in microcavities incorporating layers or quantum wells (QWs) doped with magnetic ions such as Mn^2+^ (see also the Supporting Information). The Mn^2+^ doping enhances the exciton splitting in the magnetic field due to the s,p–d exchange interaction between the spins of the electrons in the d-shell of the Mn^2+^ ion and the spins of the electron and hole forming the exciton.^20,21^ The unique ability to efficiently manipulate the excitonic component energy and spin enables strong tunability of semimagnetic polaritons by a magnetic field.^18,19,22−24^ New effects predicted to result from the coupling with the magnetic ion include effective attraction between polaritons, leading to real space self-localization^25^ and nonequilibrium self-trapping^26^ due to the magnetic polaron effect or generation of a polaritonic topological Berry phase.^27^

Here, we demonstrate semimagnetic polaritons of a *hybrid* type, obtained thanks to the strong coupling of excitons confined in magnetic QWs (MQW) with excitons in nonmagnetic QWs (NMQW) and an optical mode of a microcavity. The hybridization takes place over a distance of the order of 100 nm, which exceeds the range of tens of nanometers relevant for a dipolar polariton^28−30^ or exciton quantum tunnelling regime.^31−33^ Thanks to the optical-mode mediated coupling, the susceptibility of the excitons in the MQW to the magnetic field is extended over all hybrid polariton states arising in the structure. We perform optical spectroscopy measurements in magnetic field (see also Supporting Information) to show a continuous tuning of such properties of the hybrid semimagnetic polariton as coupling strength between the excitons and the microcavity photon, the degree of MQW and NMQW exciton hybridization, and the ratio of the excitonic to the photonic component. The coupling enables energy transfer between the MQW and NMQW, with direction and dynamics controlled by the magnetic field.

The reported polariton-mediated hybridization of a semimagnetic exciton with an exciton in a material that is nominally nonmagnetic is highly promising for the fabrication of organic, perovskite, or transition metal dichalcogenide (TMD)-based systems with enhanced magnetic properties. In particular, the ability for *in situ* tuning of their optical constants or control of nonlinear behavior by the magnetic field should become possible. Doping with magnetic ions, a standard way of enhancing the magnetic susceptibility and imposing semimagnetic properties of the semiconductor,^21^ remains inefficient and has not yet been proven in the case of these materials. At the same time, organic, perovskite, and TMD semiconductors are characterized by large oscillator strength of the exciton, which gives them the ability of strongly coupling with light.^34−38^ The single optical microcavity geometry considered in the present work is more advantageous for possible applications of hybrid semimagnetic polaritons in optoelectronics^39,40^ than the multilayered, technologically demanding, coupled microcavity one.

## Sample and Experiment

**Figure 1:**
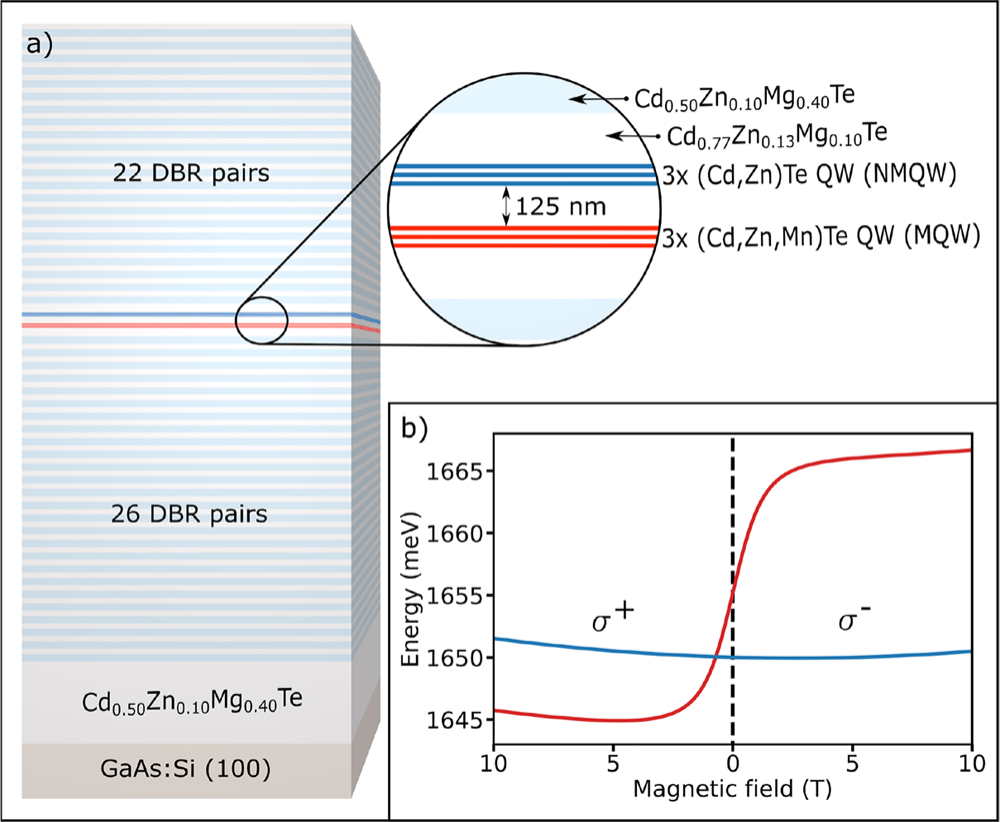
(a) Schematic view of a sample showing the composition of the layers and a close-up of the microcavity layer. (b) Energy of an exciton in a manganese doped QW (MQW, red line) and an undoped QW (NMQW, blue line) as a function of magnetic field and polarization of the light.

For the present study, we designed and fabricated a sample where two sets of QWs are placed in an optical microcavity (see the schematic view in Figure 1a). The wells in the MQW set are doped with manganese, which leads to a giant Zeeman effect on excitons when a magnetic field is applied.^19,20^ In the NMQW the exciton splitting is much smaller. This gives us the possibility to shift the exciton energy in the MQW below or above the exciton energy in the NMQW, with a crossing occurring at approximately *B* = 1 T in polarization σ^+^ (see Figure 1b).

The sample was grown by molecular beam epitaxy on a (100) GaAs:Si substrate followed by a 1 μm thick Cd_0.50_Zn_0.10_Mg_0.40_Te buffer layer. It contains a Cd_0.77_Zn_0.13_Mg_0.10_Te layer playing the role of a 3/2 λ microcavity, embedded between distributed Bragg reflectors (DBR) made of 26 and 22 pairs of Cd_0.50_Zn_0.10_Mg_0.40_Te and Cd_0.77_Zn_0.13_Mg_0.10_Te alternating layers^41^ (see Figure 1a). The microcavity is wedged, which allows for tuning of the energy of the cavity optical mode by changing the position on the sample.

The MQW and NMQW sets are located at the maxima of the electric field inside the microcavity, with a separation of 125 nm. There are three QWs in each set, and the barrier layers between them are 10 nm thick. The (Cd,Zn)Te QWs in the NMQW set are 10 nm wide, while in the MQW set the QWs are 12 nm wide and doped with 0.8% of manganese. The energy increase induced by the Mn doping is partially compensated for by the larger width of the QWs in the MQW, to reach an energy difference between MQW and NMQW excitons of around 5 meV at *B* = 0 T.

The sample was placed in a cryostat with a 10 T superconducting magnet and cooled to 1.7 K. The signal was excited and collected through a lens with a focal length of 200 mm, focusing the light into a spot of 100 μm diameter on the sample surface. The lens was mounted on XY actuators. Reflectivity mapping was done by shifting the lens in the sample plane with a step of 0.01 mm (±4 mm in each direction). For the reflectivity measurements we used a halogen lamp as a light source. For excitation of the photoluminescence a beam of wavelength 723 nm (1.715 eV) from a Mira 900 Ti:sapphire laser, operating in cw mode, was used. The power of the laser beam before entering the cryostat reached 0.66 mW. The energy of excitation is lower than the energy gap of the DBR and microcavity layers, but higher than the edge of the DBR stopband, which ensures efficient excitation of the QWs embedded in the microcavity. The detection part of the setup consisted of a 500 mm long grating monochromator (1200 gr/mm) with a Peltier cooled CCD camera on its output. For the time-resolved measurements, the laser was set to pulsed mode and a Hamamatsu streak camera with S1 photocathode mounted on a 300 mm long spectrometer detected the signal (temporal resolution better than 8 ps).

**Figure 2:**
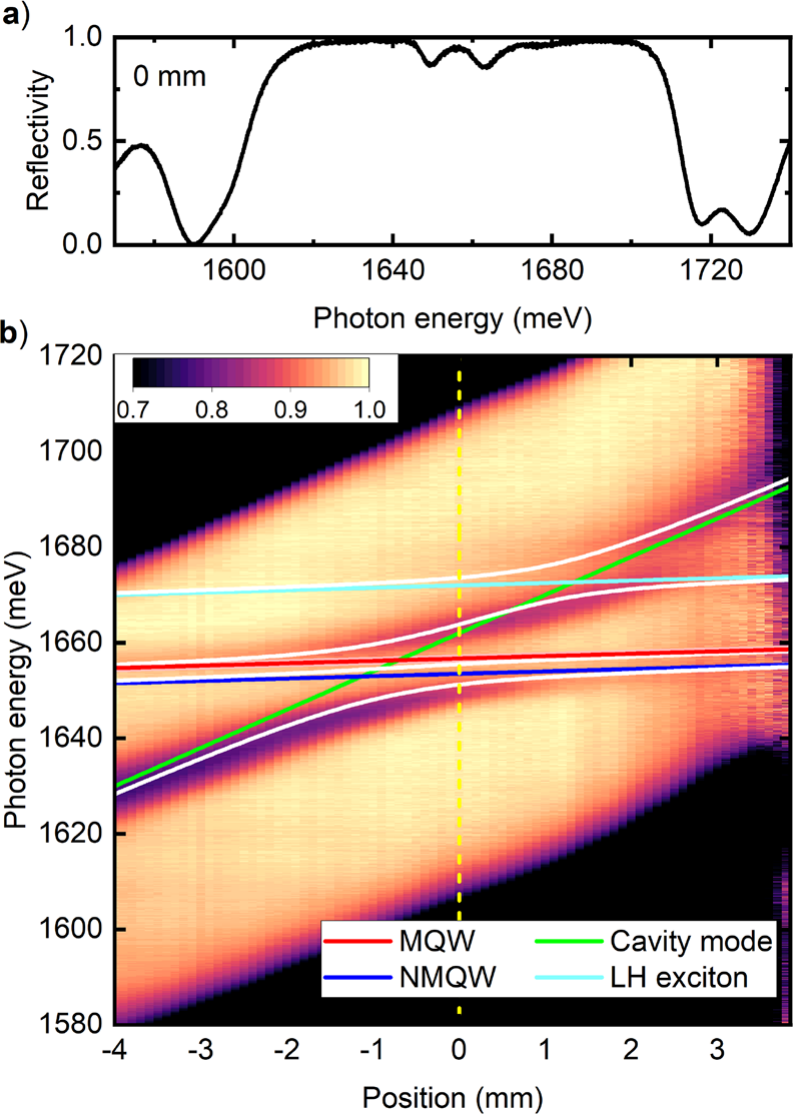
(a) Reflectivity spectrum taken at a position on the sample of *x* = 0 mm. (b) Reflectivity map as a function of the position on the sample. The anticrossing of the reflectivity minima testifies to the strong coupling conditions in the structure. The colored lines are the uncoupled energies of heavy hole excitons in MQW (red) and NMQW (blue), light hole excitons in NMQW (light blue) and the microcavity mode (green). White lines represent the calculated polariton energies.

We begin with the characterization of the studied structures by reflectivity spatial mapping. Figure 2a) shows an example reflectivity spectrum of the sample at 1.7 K. The dips superimposed on a ∼ 100 meV wide stopband represent the absorption related to exciton-polariton eigenstates of the structure. These states are composed of the microcavity mode and excitons confined in the MQW and NMQW. A map comprising a series of reflectivity spectra registered at consecutive positions on the sample along the gradient of the microcavity width is displayed in Figure 2b). The energy of the uncoupled microcavity mode increases following a linear dependence from around 1630 to 1690 meV (green line). The energies of the heavy hole (HH) exciton in the NMQW at around 1651 meV (blue line) and MQW at around 1654 meV (red line) are practically independent of the position on the sample. A signal of the light hole (LH) exciton in the NMQW at around 1670 meV is also seen (light blue line). When the excitons and the microcavity mode enter resonance an anticrossing is observed, which is an indication of strong coupling conditions and the formation of hybrid semimagnetic polariton states in the studied structure.

To describe the energies of the polariton levels, we introduce a Hamiltonian *Ĥ*, which takes the following form:^5,42,43^
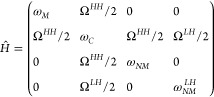
1

The energies of the HH excitons in the MQW and NMQW are denoted by ω_*M*_ and ω_*NM*_ respectively, while ω_NM_^LH^ represents the LH exciton in the NMQW and ω_*C*_ the microcavity mode. The coupling strength between the HH or LH excitons and the mode is denoted by Ω^*HH*^ or Ω^*LH*^ respectively. By fitting the eigenvalues of the Hamiltonian *Ĥ* to the minima of the reflectivity map in Figure 2b), we find that the coupling constants Ω^*HH*^ and Ω^*LH*^ take a common value of 8 ± 0.5 meV. The eigenvalues of *Ĥ* are plotted as white lines in Figure 2b).

**Figure 3:**
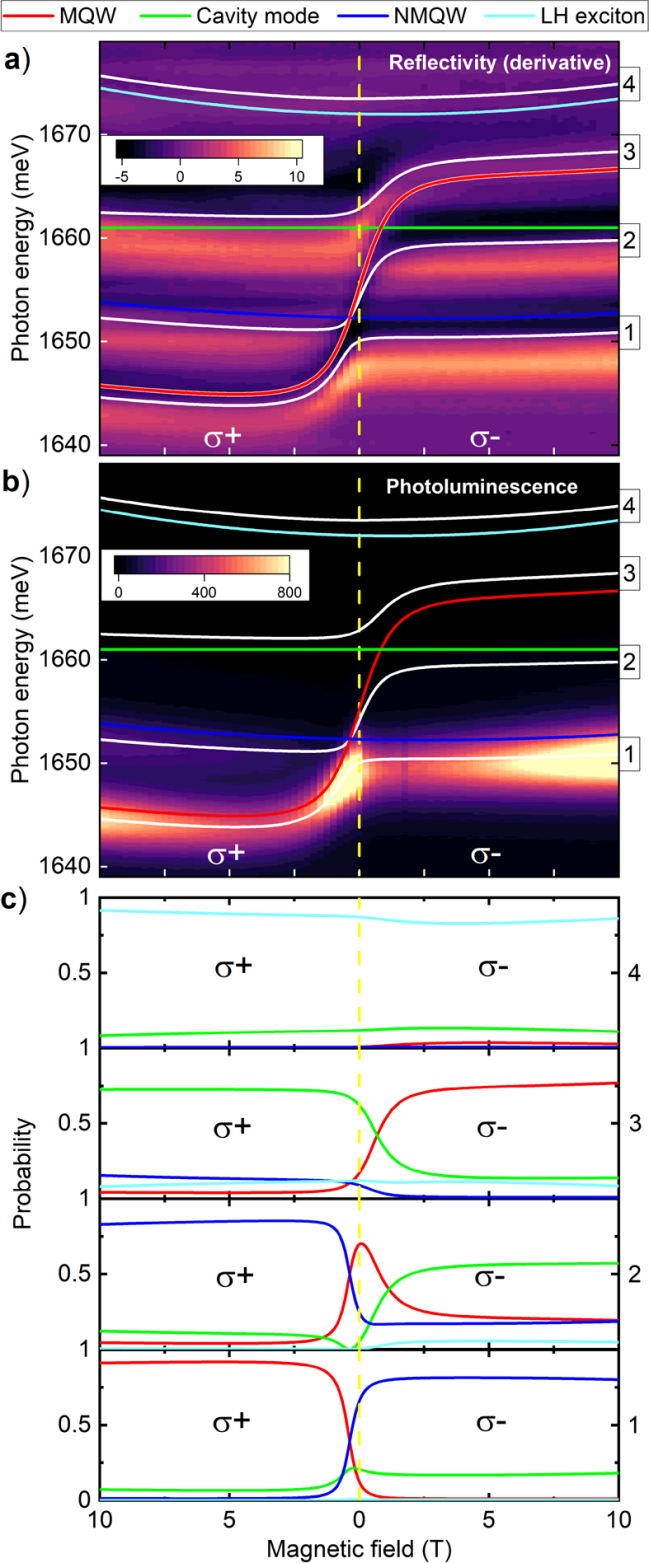
(a) Reflectivity, (b) photoluminescence, and (c) Hopfield coefficients for polariton states labeled with numbers from 1 to 4 in panels a and b, as a function of the magnetic field. To increase the visibility, the derivative of the signal is presented in panel a; the optical transitions lie between the positive (bright) and negative (dark) values on the plot. The color code is common to the whole Figure: uncoupled states of HH excitons in MQW are indicated with red, HH excitons in NMQW with blue, LH excitons with light blue and the cavity mode with green lines. White lines are the eigenvalues of the Hamiltonian *Ĥ* (eq 1) fitted to the polariton transitions in the reflectivity map shown in panel a.

In order to demonstrate the tunability of our hybrid semimagnetic polaritons we performed measurements as a function of the magnetic field at a position on the sample of *x* = 0 mm (see Figure 3), where the contributions from the NMQW and MQW excitons and the microcavity mode to the two lowest-energy polariton states are comparable. In Figure 3a) we present magneto-reflectivity and in Figure 3b) magneto-PL spectra. Figure 3c) displays the Hopfield coefficients^44^ describing the content of excitons from the MQW and the NMQW, as well as the microcavity mode in the four polariton states emerging in the structure. The Hopfield coefficients are obtained by calculation of the magnitude of the eigenvector coefficients for each polariton state.

The magneto-reflectivity measurement enables us to show the impact of the magnetic field on the energies of hybrid semimagnetic polaritons. Figure 3a) shows a Brillouin-like dependence of the optical transitions on the magnetic field, which manifests the contribution to the polariton states from the MQW excitons. Moreover, the observed anticrossings of the polariton levels confirm strong coupling conditions in the studied structure. To describe the energy dependencies observed in Figure 3a), we apply the Hamiltonian *Ĥ* (eq 1), this time taking into account the dependence of the uncoupled exciton energies on the magnetic field. We assume that the variation of the energy of the MQW exciton ω_*M*_ with the field is defined by the sum of the giant Zeeman effect described by the Brillouin function,^20^ the linear Zeeman effect (Landé factor *g* = 0.9),^45^ and the diamagnetic shift. The respective variation of the ω_*NM*_ and the ω_*NM*_^*LH*^ is the sum of the linear Zeeman effect (*g* = 0.9) and the diamagnetic shift. We assume that for the HH excitons the diamagnetic shift is equal to 0.01 eV/T^2^ and for the LH excitons it is 0.02 eV/T^2^. We consider the mode energy ω_*C*_ as independent of the field. The eigenvalues of the Hamiltonian *Ĥ* obtained as a result of fitting the data are shown in Figure 3 as white lines. The values of the coupling constants obtained from the fit are Ω_*HH*_ = Ω_*LH*_ = (8.0 ± 0.4) meV, in full agreement with those obtained from the reflectivity spatial mapping presented in Figure 2.

The prominent anticrossing at around *B* = 1 T at σ^+^ polarization provides evidence for polariton-mediated interaction between the excitons confined in the MQW and the NMQW. As revealed by the Hopfield coefficients shown in Figure 3c), the interaction leads to hybridization of the MQW and the NMQW excitonic states taking place over a distance separating the MQW and the NMQW of 125 nm. This enables us to highlight the advantage of the present structure with respect to previous works. Previously, polariton-mediated coupling of the spatially separated emitters has been conditioned by their spectral resonance.^2−11^ Here, we can tune and maximize the coupling strength of excitons in NMQW and MQW by controlling of their mutual energy overlap using magnetic field. Thanks to the Mn^2+^ doping and a resulting giant enhancement of Zeeman splitting of the exciton in the MQW, the magnetic field tunes the energy of MQW exciton with respect to NMQW exciton in the range higher for an order of magnitude than the excitons spectral line width. In consequence excitons, which in the absence of magnetic field exhibit a different energy, can be efficiently tuned to resonance. In turn, the energy overlap of the excitons and the mode is ensured by wedge-like type microcavity design and a possibility of shifting of the microcavity mode energy by selecting the spatial position on the sample.

Consistently, the magnetic field enables continuous tuning of the relative content of the NMQW and MQW excitons in the studied hybrid polariton states. In particular, for a magnetic field above the crossing at ∼1 T in polarization σ^+^, the main contribution to the lowest level “1” comes from the MQW. For other values of magnetic field and polarization the contribution to level “1” from the NMQW prevails. The possibility of tuning the energy and relative content of the NMQW and MQW excitons in the polariton levels enables us to employ the hybrid semimagnetic polaritons for the transfer of excitation between the NMQW and MQW controlled by the magnetic field. As shown in the PL spectra in Figure 3b), the emission related to the polariton state of level “1” dominates over the negligible emission from the second lowest (“2”) polariton level. For magnetic fields above the anticrossing at around 1 T in σ^+^ polarization a relaxation from state “2” to state “1” is associated with a strong increase in the MQW exciton content in the hybrid polariton. Thus, the dominating intensity of level “1” points toward a transfer of excitation from the NMQW to the MQW. The electron–hole pairs are photocreated in both quantum wells with practically the same efficiency, thus if there were no energy transfer between the MQW and NMQW, the “1” and “2” levels should exhibit a comparable emission intensity. When the MQW exciton is higher in energy than the NMQW exciton in the remaining range of fields and polarization, the direction of the transfer is reversed. We note that Zeeman splitting of the the NMQW exciton in the magnetic field is low (around 1 meV at *B* = 10 T, less than the NMQW exciton line width), and thus, the effects observed in the emission cannot be explained by magnetic field induced exciton polarization in the NMQW.

**Figure 4:**
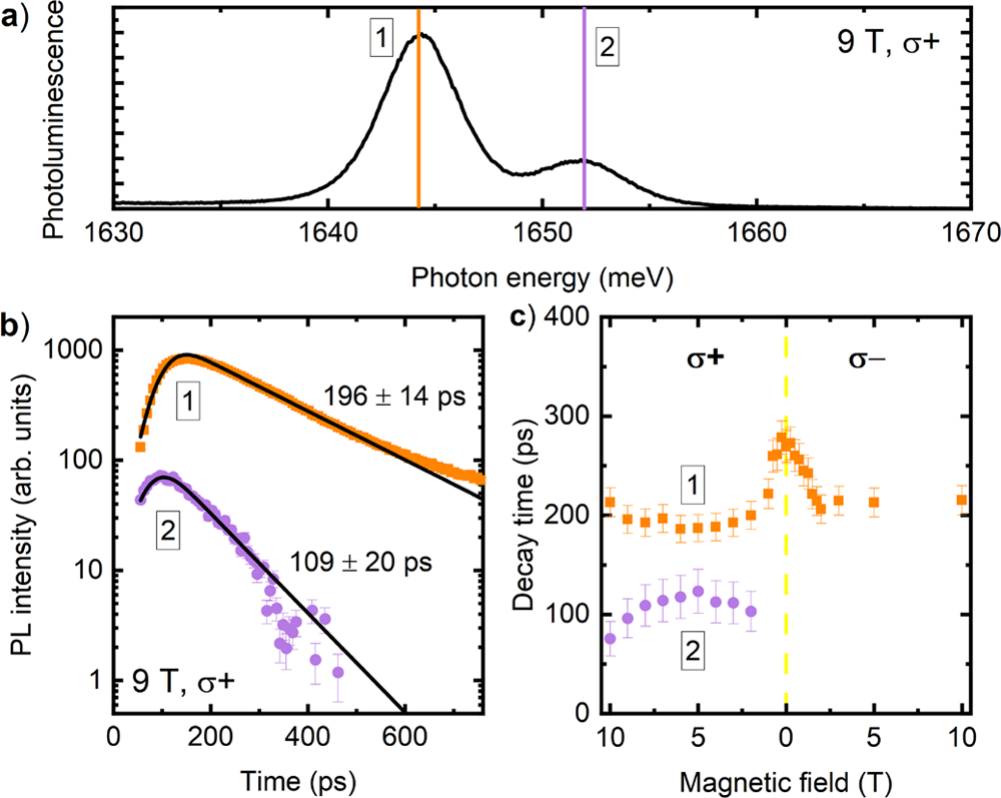
(a) Time-integrated, σ + polarized photoluminescence spectrum at *B* = 9 T, acquired using a streak camera. Transitions of “1” and “2” polariton states are in evidence. (b) Emission dynamics of polaritonic states “1” (orange points) and “2” (violet points) in σ + polarization at *B* = 9 T. Solid lines represent a fit by an exponential decay convoluted with a Gaussian curve. Decay times are indicated. (c) Decay times determined for states “1” and “2” as a function of magnetic field for two circular polarizations of the light.

In order to determine the impact of the magnetic field on the emission dynamics of the hybrid semimagnetic polaritons, we performed time-resolved PL measurements under pulsed excitation. An example of a time-integrated emission spectrum taken at *B* = 9 T in σ^+^ polarization is shown in Figure 4a). The spectrum contains transitions of the polariton states “1” and “2” at 1644 and 1652 meV respectively. For each value of the magnetic field varied with a step of 0.25 T between 0 and 10 T we fitted a sum of two Gaussian curves to the spectra in consecutive delays following the excitation pulse, determining the intensity of the transitions “1” and “2” as a function of time. Next, we fitted an exponential decay convoluted with a Gaussian curve (40 ps wide) to the obtained intensities vs time (see Figure 4b) for sample dependencies). Decay time constants determined in this way are shown in Figure 4c). For fields above 1 T in σ^+^ polarization, the determination of the state “2” lifetime is not possible, since its emission is too weak due to efficient polariton relaxation to the lowest state “1”.

Several processes affect the emission dynamics in the studied system, such as radiative and nonradiative recombination, intra- and interwell transfer and spin flip of excitons forming the polaritons, as well as acceleration of the emission due to the coupling with the mode, which makes interpretation of the collected time-resolved data not as straightforward. In a simplified picture, the decay time of the lowest polaritonic state “1” reflects the lifetime of this state prolonged by a transfer of polaritons from higher energy states. In turn, the decay time of state “2” basically reflects the state “2” lifetime shortened by the transfer of the polariton population from state “2” to state “1”. Consistently, the decay time of state “1” sets the upper, while the decay of state “2” the lower limit for the relaxation time from the “2” to ”1” polariton level, hence the transfer of energy between the MQW and NMQW. The results presented in Figure 4c therefore indicate that the transfer time remains roughly in the range between 100 and 280 ps. The maximum of the decay time of state “1” in the vicinity of *B* = 0 T, in conjunction with the respective maximum of the emission intensity (see Figure 3b) suggests that the transfer between the MQW and NMQW is most efficient when the degree of NMQW and MQW exciton hybridization is a maximum (see the Hopfield coefficients for level “1” in Figure 3c).

We have presented hybrid semimagnetic polaritons emerging as a result of the coupling of an optical microcavity mode with excitons in semimagnetic and nonmagnetic quantum wells separated by 125 nm. The doping of the MQW with Mn^2+^ ions enables the energies of the MQW excitons to be shifted below or above those in the NMQW using an external magnetic field thanks to the giant Zeeman effect. This provides an efficient tool for control of the degree of hybridization of the excitons in the MQW and NMQW and the properties of the emerging polariton states. The strong coupling and hybridization enable a transfer of energy between the MQW and NMQW excitonic states, occurring with a time constant of the order of 100 ps. The transfer direction is alterable by the magnetic field, which might be further exploited in such diverse applications as information processing in spintronic or optoelectronic devices or energy harvesting in semiconductor-based solar cells. The demonstrated polariton-mediated hybridization of the semimagnetic exciton with the exciton in a nominally nonmagnetic, spatially separated layer holds prospects for the fabrication of magnetically controlled devices exploiting organic, perovskite, or dichalcogenide semiconductors.

## Supporting Information Available

Exciton–polaritons, diluted magnetic semiconductors, and reflectivity of the studied structure (PDF)
