# Supplementary material for: Hybrid Semimagnetic Polaritons in a Strongly Coupled Optical Microcavity
Source: J Phys Chem Lett. 2021 Aug 5;12(31):7619–24. doi: 10.1021/acs.jpclett.1c01894 (PMC8397346; doi:10.1021/acs.jpclett.1c01894)
Supplement: Supplementary file 1 — jz1c01894_si_001.pdf [file jz1c01894_si_001.pdf]

Supplementary material to: Hybrid semimagnetic polaritons in a strongly coupled optical microcavity

Tomasz Fąs, Maciej Ściesiek, Wojciech Pacuski, Andrzej Golnik, and Jan Suffczyński*

Institute of Experimental Physics, Faculty of Physics, University of Warsaw, 5 Pasteura St., 02-093 Warsaw, Poland

E-mail: j.suffczynski@uw.edu.pl

Exciton-polaritons

When kinetic energy of an electron excited from the valence to conduction band of a semiconductor is low enough, a bound electron-hole state called an exciton is formed (see Suppl. Fig. 1). Exciton binding energy varies from the order of a single meV in the case of GaAs up to 1 eV in the case of organic semiconductors. Radiative recombination of the exciton releases a photon carrying exciton energy.

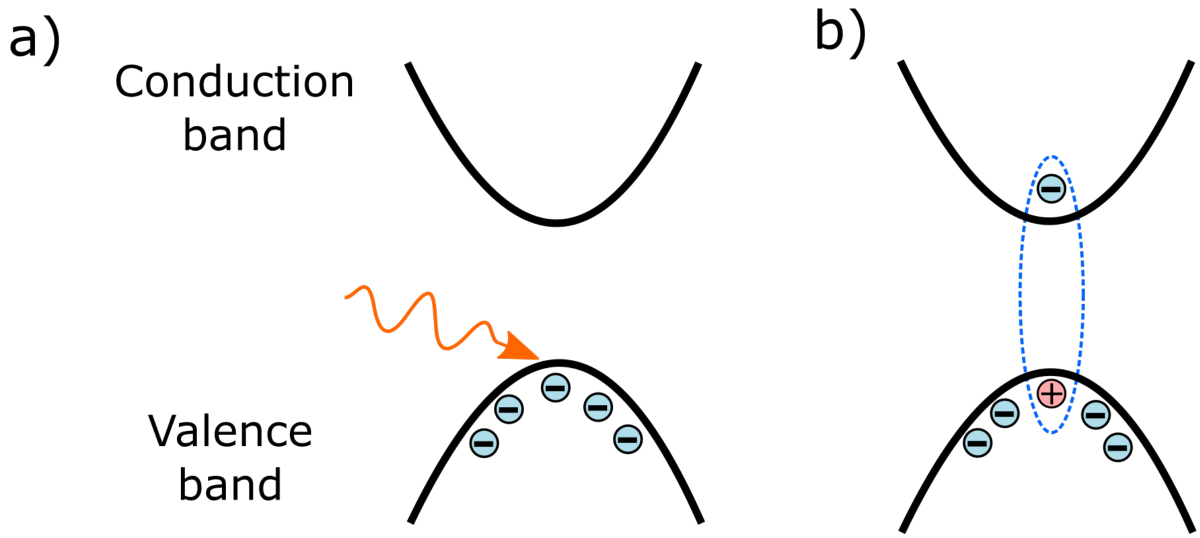

Figure 1: Schematic representation of an exciton. a) A photon (orange line) excites an electron from the conduction band leaving a hole in a valence band. b) Coulomb interaction between electron and hole leads to formation of a hydrogen-like quasiparticle called exciton.

When a semiconductor layer is inserted into a resonant optical microcavity, the exciton couples to the microcavity optical mode. If the rate of energy dissipation from the microcavity is lower then the rate at which exciton and optical mode exchange the energy, a strong light-matter coupling regime conditions are met. In this case the photon emitted in exciton recombination is re-absorbed and re-emitted several times until it leaves the microcavity. Such coupling results in the emergence of a new quasiparticle called an exciton-polariton or just polariton. Being a quantum superposition of the light and matter, the polariton inherits the properties of both of its constituents.

Semiconductor optical microcavity typically utilizes Distributed Bragg Reflectors (DBR)

as mirrors. The DBR is a structure consisting of alternating layers of two materials with thicknesses of d_i and refractive indices n_i $(i = 1, 2)$, with the condition $d_i n_i = \lambda/4$, where λ is a wavelength for which the mirror is designed. A schematic of such a microcavity is shown in Fig. 2a). A semiconductor quantum well (QW) confining excitons is placed in an antinode of a standing electromagnetic wave confined within the microcavity surrounded by the DBRs.

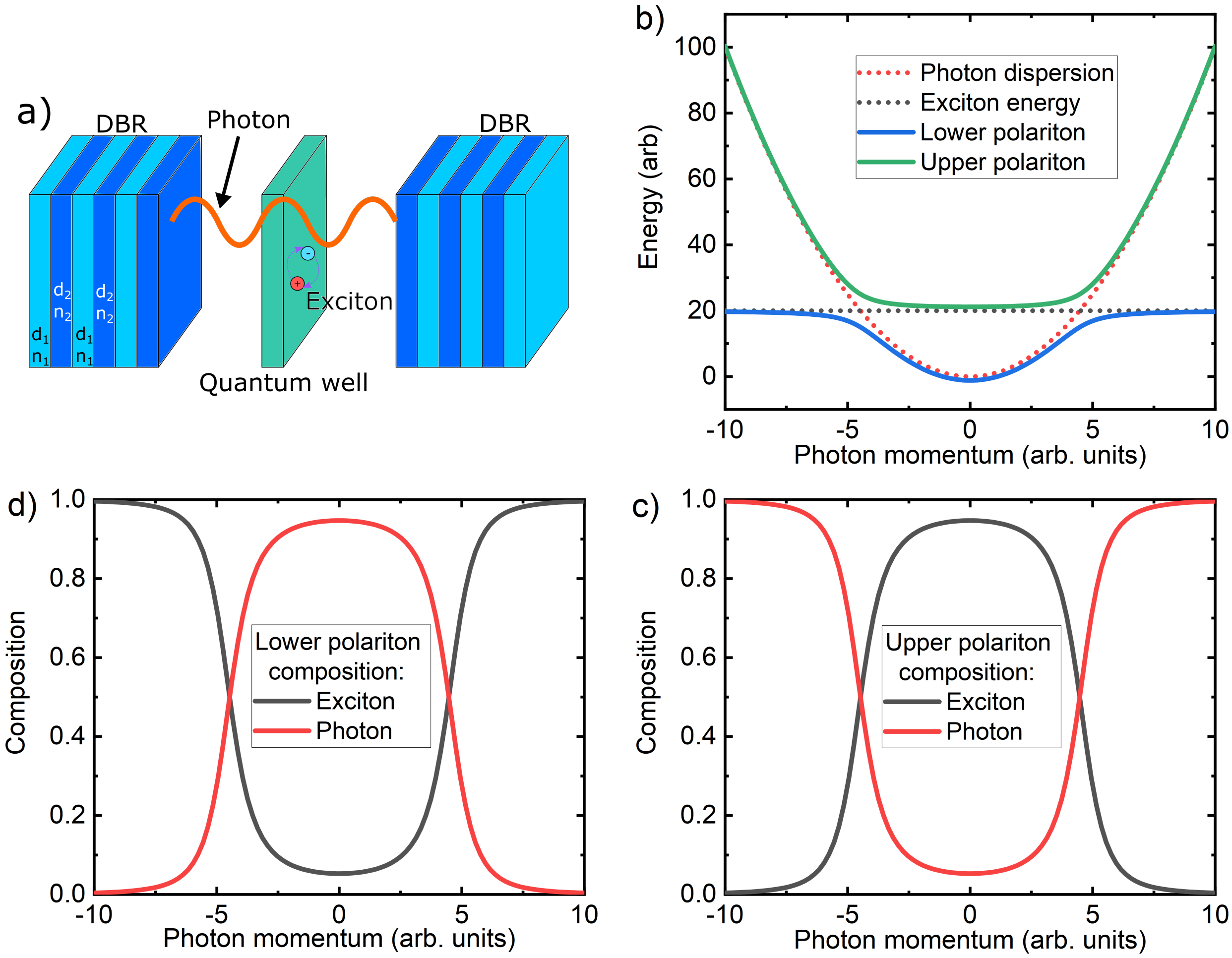

Figure 2: a) A schematic representation of an optical microcavity, with DBRs playing a role of mirrors. A quantum well layer confining excitons placed inside the microcavity is indicated. b) A schematic plot showing energies of exciton, optical mode and upper and lower polariton levels as a function of the wavevector directed in-plane of the microcavity. Polariton energies are calculated using Hamiltonian H_0. An anticrossing of levels is a characteristic feature testifying the presence of strong coupling conditions. c) and d) Hopfield coefficients of respectively the upper and lower polariton level.

The system can be described by a simple Hamiltonian, consisting of energy of the exciton E_{ex}, energy of the photon E_{ph} and the coupling constant Ω that describes the coupling strength between the exciton and photon. The matrix form of such Hamiltonian is as follows:

$$\hat{H}_0 = \begin{pmatrix} E_{ex} & \Omega \\ \Omega & E_{ph} \end{pmatrix} \tag{1}$$

The eigenvalues of the Hamiltonian H_0 describe the energies of the emerging two polariton states, while the eigenvectors describe the polariton's wavefunction. The eigenvector $|\Psi\rangle$ can be decomposed in the uncoupled states basis as: $|\Psi\rangle = \alpha|EX\rangle + \beta|PH\rangle$, where $|EX\rangle$ represents the exciton and $|PH\rangle$ represents the photon. Coefficients α and β in the polariton context are named Hopfield coefficients.[1] As we see, squares of a modulus of Hopfield coefficients describe the superposition of uncoupled exciton and photon states in the polariton.

The energy of photon confined in the microcavity is described by the approximately quadratic-like relation with respect to the its momentum in plane of the microcavity.[2] In the case of the exciton, the effective mass is large enough to justify assumption of a flat exciton dispersion and a constant exciton energy at each of quantum well confined levels. Determination of the eigenvalues of the Hamiltonian H_0 (Eq 1) yields the polariton energies shown in Fig. 2b). In this case, we used $\Omega = 5$ and $E_{ex} = 20$ in arbitrary units. The red and gray curves represent the exciton and photon energies, respectively, while the blue and green curves represent the energies of two polariton levels. They are referred to as the lower and the upper in terms of their energy values.

The squares of Hopfiled coefficients as a function of the in-plane photon momentum are shown in Fig. 2c) and d) for the upper and the lower polariton level, respectively. As can be seen, in the considered example case the lower polariton at large positive detunings (being the energy difference between the mode and the exciton) the lower polariton is almost entirely composed of the exciton, whereas the upper polariton is almost entirely composed of the

photon. At zero detuning, i.e., where QW-confined exciton and microcavity-confined photon are in resonance, both polariton states contain equal contributions from both uncoupled constituents. At negative detuning the lower polariton is mostly of photonic, while the upper polariton of excitonic nature.

By placing two or more different emitters at the antinodes of the electric field inside the microcavity we create polariton states being a superposition of multiple excitons and a microcavity mode. Such superposition is possible thanks to the coupling of different emitters (quantum wells in the case of the present work) to a common optical mode. The polaritons created in such manner are named *hybrid* polaritons, to emphesize their multiple exciton composition.

Diluted Magnetic Semiconductors

A diluted magnetic semiconductor (DMS) is a material obtained on the basis of a standard semiconductor in which a fraction of the cations has been randomly replaced with ions with a non-vanishing magnetic moment, as illustrated in Suppl. Fig. 3a). An example might be CdTe or, studied in the present work, ZnTe doped with Mn^{2+} ions (Mn^{2+} with its five electrons occupying d-shell it exhibits spin of 5/2). Localized spins of magnetic ions couple through *s,p-d* interaction with the spins of spatially delocalized band carriers.[3,4] As a result, the external magnetic field is effectively enhanced in the semiconductor, acting on carriers through the paragenetically ordered magnetic dopants. This leads to such magnetic field induced phenomena as giant Zeeman splitting of bands or giant magneto-optical Kerr effect.[3]

Orders of magnitude enhanced valence and conduction band splitting due to the giant Zeeman effect induces giant exciton levels splitting. When considering Faraday configuration (magnetic field parallel to the light propagation direction), as in the present work, the selection rules governing the emission and absorption of light by excitonic levels allow only

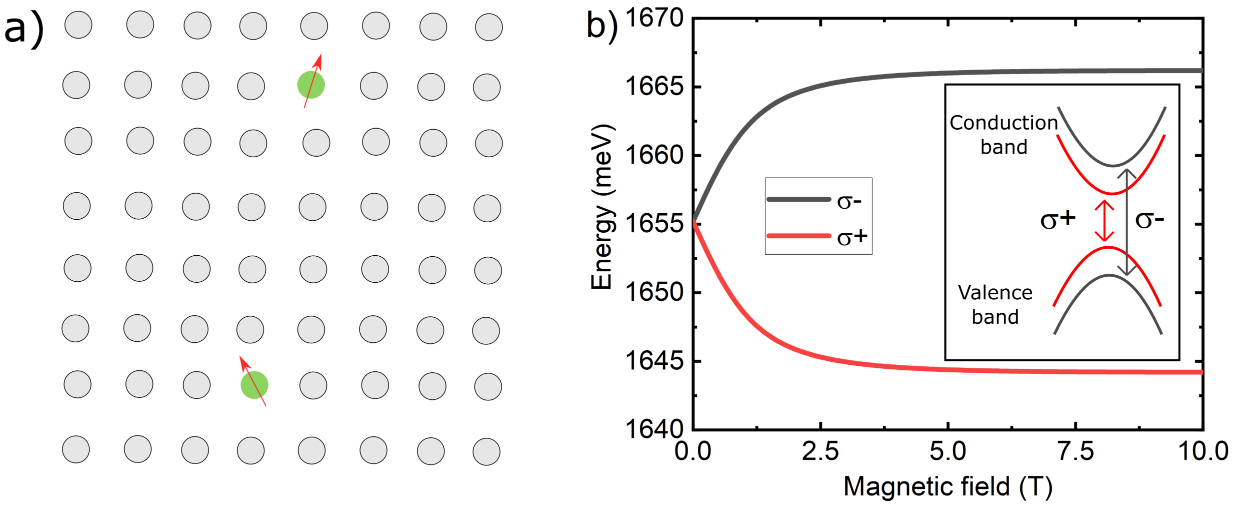

Figure 3: a) In a diluted magnetic semiconductor (DMS), a part of cations in replaced with magnetic ions (green dots). Those ions interact with charge carriers through *s,p-d* interaction, enhancing the Zeeman splitting of the valence and conduction band, so that of the excitons. b) In the case of Mn^{2+} doped ZnTe, the exciton splitting follows a Brillouin function, as shown. Inset: optical selection rules imply respectively $\sigma+$ or $\sigma-$ polarization of the optical transitions shifting towards lower or higher energy in magnetic field.

for a circularly polarized light to be emitted/absorbed. The splitting between those levels changes as a function of magnetic field follows so called Brillouin function.[3] The schematic representation of conduction and valence band splitting, the selection rules and the shape of Brillouin function is presented in Suppl. Fig. 3b). The Brillouin function itself is described as follows:

$$B_J(x) = \frac{2J+1}{2J} \coth\left(\frac{2J+1}{2J}x\right) - \frac{1}{2J} \coth\left(\frac{1}{2J}x\right) \tag{2}$$

where J is the total quantum angular momentum of magnetic ions and $x = g_{DMS}\mu_B JB/k_B T$, with g_{DMS} being the g-factor of a given DMS, k_B - Boltzmann constant, μ_B - Bohr magneton, T - effective temperature of the material, and B is the magnetic field strength.[3,5]

Reflectivity of the studied structure

In our paper, we employ reflectivity measurements to determine the energies of the polariton states in the structure, where two different quantum wells are coupled to a common mode of a microcavity. In our reflectivity measurement, a beam of white, unpolarized light produced by a halogen lamp incidents on the structure surface. Absorption of the light is possible only at the energies specific to polariton states, showing up as dips in the reflectivity spectra. In that way, the measurement enables determination of the polariton states' energy by tracing the energy of those dips, for example as a function of such parameters as spatial position on the sample or the strength of external magnetic field.

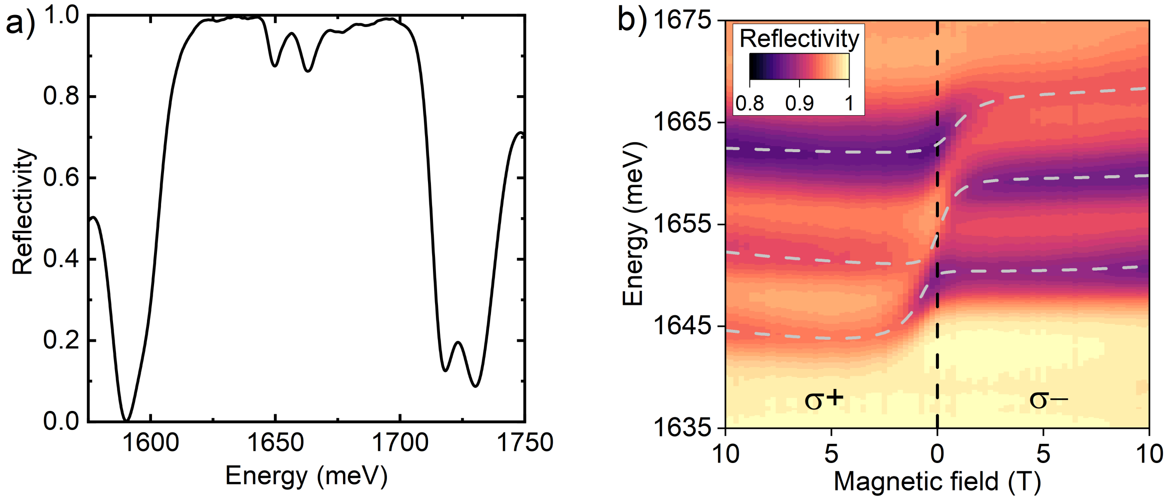

Figure 4: a) Reflectivity spectrum of the studied sample in the absence of external magnetic field. High reflectivity region called a *stopband* is present in the central part of the spectrum. A number of minima are superimposed in the stopband, hinting an existence of polariton states inside a microcavity. b) Reflectivity spectra taken as a function of magnetic field for two circular polarizations of the light. Grey line highlights the position of the reflectivity minima. Anticrossing of levels observed at around 1 T in $\sigma+$ polarization provides a proof of strong coupling conditions in the sample and formation of polariton states.

An example reflectivity spectrum of the studied sample is shown Suppl. Fig. 4a). We can discern two distinct and two weaker minima. The minima indicate the presence of four states that absorb the incident light. In order to proof that the minima indeed represent polaritons, thus mutually coupled states, we should change energy of the mode or one of the

excitonic states and trace evolution of energy of the minima in the spectrum. In the main article's Fig. 2 we changed the microcavity mode energy by shifting the beam position on a sample. As a result, we were able to evidence the reflectivity minima anticrossing, providing a clear indication of strong coupling and emergence of polariton states. We perform a reflectivity measurement as function of magnetic field in Suppl. Fig. 4b) to induce change of the energy of the exciton confined in the quantum well doped with Mn^{2+} ions. We refer to this measurement as a "magneto-reflectivity measurement". We see anticrossings and Brillouin-like behavior of all the reflectivity minima indicating that a strong susceptibility of one of the uncoupled excitonic states to the magnetic field is extended over all states. Such coupling indicate that these are polariton states.

References

(1) Hopfield, J. J. Theory of the Contribution of Excitons to the Complex Dielectric Constant of Crystals. *Phys. Rev.* **1958**, *112*, 1555–1567.

(2) Kavokin, A.; Baumberg, J. J.; Malpuech, G.; Laussy, F. P. *Microcavities*; Oxford University Press, Inc.: New York, NY, USA, 2008.

(3) Furdyna, J. K. Diluted magnetic semiconductors. *Journal of Applied Physics* **1988**, *64*, R29–R64.

(4) Gaj, J.; Planel, R.; Fishman, G. Relation of magneto-optical properties of free excitons to spin alignment of Mn2+ ions in Cd1-xMnxTe. *Solid State Communications* **1979**, *29*, 435 – 438.

(5) Kittel, C. *Introduction to Solid State Physics*, 8th ed.; Wiley, 2004.
